# Associations between perceptions of care and women’s childbirth experience: a population-based cross-sectional study in Rwanda

**DOI:** 10.1186/s12884-017-1363-z

**Published:** 2017-06-09

**Authors:** Judith U. Mukamurigo, Marie Berg, Joseph Ntaganira, Laetitia Nyirazinyoye, Anna Dencker

**Affiliations:** 1 Institute of Health and Care Sciences, Sahlgrenska Academy, University of Gothenburg, Box, 457, 405 30 Gothenburg, Sweden; 2 College of Medicine and Health Sciences, School of Public Health, University of Rwanda, Kigali, Rwanda; 3 Centre for Person-Centred Care (GPCC), Sahlgrenska Academy, University of Gothenburg, Gothenburg, Sweden

**Keywords:** Childbirth, Experience, Women, Quality of care, Rwanda

## Abstract

**Background:**

In recent years Rwanda has achieved remarkable improvement in quality of maternity care services but there is evidence of deficiencies in care quality in terms of disrespectful care. Women’s overall childbirth experience is an important outcome of childbirth and a factor in assessing quality of care. The aim of this study was to investigate how women’s overall childbirth experience in Rwanda was related to their perceptions of childbirth care.

**Methods:**

A cross-sectional household study of women who had given birth 1–13 months earlier (*n* = 921) was performed in the Northern Province and in the capital city. Data was collected via structured interviews following a questionnaire. Significant variables measuring perceptions of care were included in a stepwise forward selection logistic regression model with overall childbirth experience as a dichotomised target variable to find independent predictors of a good childbirth experience.

**Results:**

The majority of women (77.5%) reported a good overall childbirth experience. In a logistic regression model five factors of perceived care were significant independent predictors of a good experience: confidence in staff (Adjusted OR 1.73, 95% CI 1.20–2.49), receiving enough information (AOR 1.44, 95% CI 1.03–2.00), being treated with respect (AOR 1.69, 95% CI 1.18–2.43), getting support from staff (AOR 1.75, 95% CI 1.20–2.56), and having the baby skin-to-skin after birth (AOR 2.21, 95% CI 1.52–3.19).

**Conclusions:**

To further improve childbirth care in Rwanda and care for women according to their preferences, it is important to make sure that the childbirth care includes the following quality aspects in national and clinical guidelines: build confidence, provide good information, treat women and families with respect, provide good professional support during childbirth and put the newborn baby skin-to-skin with its mother early after birth.

## Background

In recent years Rwanda has achieved remarkable improvement in quality of maternity care services [1, 2]. Almost all pregnant women (99%) attend antenatal care at least once during pregnancy, although only 47% had their first antenatal care visit during the first trimester as recommended. Furthermore, health-facility-based childbirth assistance by skilled care providers has increased from 69% in 2010 to more than 90% in 2014. The majority of women with uncomplicated pregnancy give birth at a health centre, while pregnant women with complications are referred to a district hospital or a tertiary hospital according to severity of complication [3].

The quality progress might be attributed to the introduction of health insurance and Community Health Workers (CHW) who are sufficiently available and motivated. In each village in Rwanda, volunteers are elected to act as CHWs. There are two general CHWs – one male and one female (called a *binome*) who are responsible for community health, nutrition, and HIV/AIDS prevention – and a maternal health worker (referred to as an *Animateur de Santé Maternelle*), who manages infants, and pre- and postnatal maternity care. In addition, each village has a CHW in charge of social affairs who is dedicated to addressing the wellbeing of individuals and the community [4, 5].

An important aspect of maternity care involves exploring women’s views [6] but few studies have been conducted in low-income countries. An observational study in five African countries, including Rwanda, found that poor quality of maternity care was related to poor interactions between women and care providers and also to a lack of information provided to the women [7]. Another study, in Nigeria, found that women often were subjected to disrespectful and abusive treatment as part of their childbirth experiences [8].

A positive childbirth experience is important for the woman’s wellbeing, facilitates the mother-child bonding and may have implications for the future health for both the mother and baby [9–11]. On the contrary, a negative experience increases the risk for postpartum depression, secondary fear of childbirth [12] and post-traumatic stress disorder [13]. Despite progress in Rwanda in achieving universal access to reproductive health and maternity services, there is evidence of deficiencies in health care quality [14], and to our knowledge no study has focused on women’s experiences of childbirth in Rwandan health facilities. Therefore, the aim of this study was to investigate how women’s perceptions of care received during labour and birth in Rwanda were related to their overall childbirth experience.

This study is part of the Maternal Health Research Programme in Rwanda (MatHeR) undertaken by the University of Rwanda in collaboration with the University of Gothenburg and Umea University in Sweden.

## Methods

### Setting and data collection

A retrospective cross-sectional population-based household study was conducted from 7th July to 15th August 2014 in the Northern Province and Kigali City, the capital and largest city in Rwanda. From a complete list of 4791 villages in the five districts of the Northern Province and three districts within Kigali City [15] a random selection was done to select 48 villages. Approximately 20% of Rwandan population lives in urban areas and this proportion of villages were selected from urban areas [3]. A proportionate number of households were selected from each village and community health workers in each village who keep records of pregnancies and childbirths helped identify study participants. Sample size was estimated to include 922 participants from a total population of 2,865,355 and is described elsewhere [16].

### Participants

Women were eligible for inclusion if they had given birth 1 to 13 months earlier (gave birth between 31st May 2013 and 30th June 2014). In total, 922 women were asked to participate and only one woman declined. All women received verbal and written information and all participants gave written consent. The interviews were conducted in private, and only one woman was interviewed in each household for confidentiality purposes.

### Data collection

Twelve female interviewers (10 nurses/midwives and 2 clinical psychologist) were recruited to interview eligible participants. Before the data collection the interviewers were trained for 5 days; 1 day of training focused on identifying eligible households and other listing procedures, 2 days were spent on questionnaire contents and ethical issues, 1 day of fieldwork to pre-test survey instruments and fieldwork procedures and 1 day of debriefing with feedback after the pre-test fieldwork.

### Questionnaire

An interview questionnaire was made by the research team. It included background variables, questions about the women’s perceptions of care received during childbirth and a question where the women rated their overall childbirth experience. The questionnaire was developed in English and translated into Kinyarwanda by a medical doctor native in Kinyarwanda and skilled in English. The Kinyarwanda version of the questionnaire was checked during a working day with the data collectors and the research team and adjustments were done. Next a pilot study including 36 women from a neighbouring village was done to test face validity of questionnaire. All 36 women completed the test interviews and some minor changes of wording in a few questions were done but no major revision of the questionnaire was needed.

The outcome variable in this study was women’s rating of the overall childbirth experience. The question was formulated: “What was your overall experience of the childbirth?” and answered on an 11-point numeric rating scale ranging from 0 (Very bad) to 10 (Very good). In order to be used as the dependent variable in a logistic regression analysis, the response options were recoded to a dichotomous response, where 8–10 was defined as a good experience and 0–7 was considered being not a good experience (bad or mixed). The dichotomisation was based on the distribution of responses. The median value was 9 and 8–10 were the most common values with a clear decline in response rate from 8 (15.0%) to 7 (9.5%).

Used as independent variables were statements concerning perceptions of care for the woman to agree with or not; the women’s confidence in the medical staff, information received during childbirth, being treated with respect by staff, receiving necessary pain relief, getting support from the health care staff, getting help with initiating breastfeeding, and having the baby skin-to-skin after birth. These statements were answered on 4-point Likert scale with response options ranging from “Totally agree” to “Totally disagree”, except the question about having the baby skin-to-skin, which had dichotomous response options, with a “Yes” or “No”.

### Statistical analyses

Descriptive analyses were computed for background variables; age, parity, education, marital status, number of people in the household, home province, place of childbirth, mode of delivery, uncomplicated pregnancy, maternal health status, age of baby and health status of the newborn baby 1 day after birth.

Univariable analyses were performed to test the association between each of the independent variables and the dependent variable to find predictors of childbirth experience. Distributions or response options and unadjusted odds ratios (OR) with 95% confidence intervals for each independent variable against the dependent variable were computed.

Variables from the univariate analyses with a *p*-value below 0.05 were included in a stepwise forward selection logistic regression model with the dichotomised childbirth experience as the outcome variable. Adjusted odds ratios (AOR) from the multivariable logistic model for childbirth experience were calculated with their 95% confidence intervals (CI) for each significant variable.

SPSS version 23 (SPSS Inc., Chicago, IL, USA) and version 9 of SAS System for Windows (Cary, NC, USA) was used for statistical analyses. All significance tests were two-tailed.

## Results

After informed consent 921 of 922 (99.9% response rate) invited women answered the questionnaire in an interview. Of these 898 women (97.5%) rated their overall experience and could be included in the analysis. Background characteristics; age, parity, education, marital status, number of people in the household, home province, place of childbirth, mode of delivery, uncomplicated pregnancy, maternal health status, age of baby and health status of the newborn baby 1 day after birth of the total study population (*n* = 921) and those included in the analysis (*n* = 898) are presented in Table 1.

**Table 1:** Characteristics of study participants (*n* = 921) and of the women that answered the childbirth experience question (*n* = 898)

Characteristics	Total group *n* = 921	Answered overall experience question *n* = 898
	n (%)	n (%)
Age		
15–24	295 (32.1)	292 (32.6)
25–34	489 (53.2)	476 (53.1)
35–44	133 (14.5)	126 (14.0)
> 44	3 (0.3)	3 (0.3)
Mean (SD)	27.8 (6.0)	27.7 (5.9)
Median (min; max)	27.0 (15.0; 46.0)	27.0 (15.0; 46.0)
Parity		
Primiparous	326 (35.4)	319 (35.5)
Multiparous	595 (64.6)	579 (64.5)
Education		
Never attended school	76 (8.4)	75 (8.5)
Primary school	635 (70.2)	618 (70.1)
Secondary school	163 (18.0)	158 (17.9)
University level	31 (3.4)	31 (3.5)
Marital Status		
Married & cohabiting	774 (84.1)	754 (84.1)
Separated, widowed or single	146 (15.9)	143 (15.9)
Number of people in household		
1–4	472 (51.4)	461 (51.5)
5–7	345 (37.6)	338 (37.8)
> 7	101 (11.0)	96 (10.7)
Province		
Kigali	304 (33.0)	296 (33.0)
Northern Province	617 (67.0)	602 (67.0)
Health Insurance		
Community based	686 (74.6)	670 (74.7)
Public and private	46 (5.0)	45 (5.0)
No insurance	188 (20.4)	182 (20.3)
Place of childbirth		
Health centre	582 (63.3)	571 (63.7)
District hospital	230 (25.0)	227 (25.3)
Referral hospital or Private clinic	60 (6.5)	60 (6.7)
At home or on the way to the clinic	47 (5.1)	38 (4.2)
Mode of delivery		
Vaginal birth	803 (88.0)	781 (87.9)
Planned CS	33 (3.6)	33 (3.7)
Emergency CS	76 (8.3)	75 (8.4)
Complications or problems during childbirth		
No complications	765 (83.4)	745 (83.3)
Complications	152 (16.6)	149 (16.7)
Health status one day after childbirth		
Very good	50 (5.4)	48 (5.4)
Good	573 (62.4)	561 (62.5)
Neither good nor bad	224 (24.4)	217 (24.2)
Bad	59 (6.4)	58 (6.5)
Very bad	13 (1.4)	13 (1.4)
Age of baby		
1–6 months	484 (52.8)	475 (53.2)
7–13 months	432 (47.2)	418 (46.8)
New-born health status one day after birth		
Very good	442 (48.0)	429 (47.8)
Good	365 (39.6)	357 (39.8)
Neither good nor bad	51 (5.5)	49 (5.5)
Bad	49 (5.3)	49 (5.5)
Very bad	14 (1.5)	14 (1.6)

CS = Caesarean Section

Distribution of responses for the dependent outcome variable (*n* = 898) are shown in Table 2. Median rating of the overall childbirth experience was 9 with a range of answers between 0 and 10. The majority (77.5%) rated an overall experience between 8 and 10, defined as a good experience in this study.

**Table 2:** Distribution of responses to outcome variable of the logistic regression analysis, *n* = 898

What was your overall experience of the childbirth? Response options on a numeric rating scale 0–10, where 0 = Very bad and 10 = Very good	
Mean (SD)	8.56 (1.86)
Median (Range)	9 (0–10)
Good experience (8–10), n (%)	696 (77.5%)
0	7 (0.8%)
1	0 (0%)
2	5 (0.6%)
3	4 (0.4%)
4	12 (1.3%)
5	49 (5.5%)
6	40 (4.5%)
7	85 (9.5%)
8	135 (15.0%)
9	156 (17.4%)
10	405 (45.1%)

Seven variables with statements about perceptions of care (confidence in staff, receiving enough information, being treated with respect, getting enough pain relief, getting support from staff, getting help with breastfeeding and having the baby skin-to-skin after birth) showed a significant relation (*p* < 0.05) with the dichotomised outcome variable in univariable analyses. Distributions of response options for the statements among those who rated a good overall experience and *p*-values can be seen in Table 3 (*n* = 898).

**Table 3:** Variables of perceptions of childbirth care by Overall childbirth experience, *n* = 898

Quality care variable	Total (*n* = 898)	Overall childbirth experience 8–10 (*n* = 696)	*p*-value
I had confidence in the medical skills of the staff during childbirth			
Totally disagree	8	3 (37.5%)	
Mostly disagree	21	7 (33.3%)	
Mostly agree	357	231 (64.7%)	
Totally agree	498	444 (89.1%)	<.0001
I got information on what was happening during childbirth			
Totally disagree	14	5 (35.7%)	
Mostly disagree	79	40 (50.6%)	
Mostly agree	390	273 (70.0%)	
Totally agree	403	367 (91.1%)	<.0001
The health care staff treated me with respect during childbirth			
Totally disagree	20	4 (20.0%)	
Mostly disagree	24	7 (29.2.%)	
Mostly agree	368	251 (68.2%)	
Totally agree	474	423 (89.2%)	<.0001
I got the pain relief I needed during childbirth			
Totally disagree	31	17 (54.8%)	
Mostly disagree	133	94 (70.7%)	
Mostly agree	376	275 (73.1%)	
Totally agree	346	299 (86.4%)	<.0001
I got the support from the health care providers that I needed during childbirth			
Totally disagree	10	2 (20.0%)	
Mostly disagree	37	13 (35.1%)	
Mostly agree	431	302 (70.1%)	
Totally agree	407	368 (90.4%)	<.0001
The health care providers helped me to start breastfeeding			
Totally disagree	160	118 (73.7%)	
Mostly disagree	198	141 (71.1%)	
Mostly agree	201	148 (73.6%)	
Totally agree	326	277 (84.9%)	0.0006
I had my baby skin to skin after birth			
No, baby not skin to skin	248	161 (64.9%)	
Yes, baby skin to skin	638	524 (82.1%)	<.0001

**Fig. 1:**
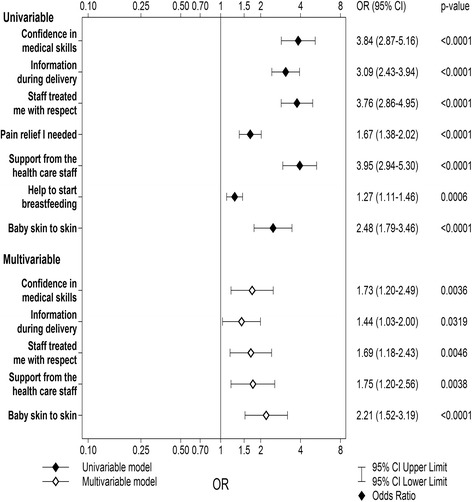
Univariable and multivariable logistic regression of perceptions of care variables against Overall childbirth experience, *n* = 898

All significant predictors from the univariate analyses were entered into a multivariate stepwise logistic regression model to find significant independent predictors of a good overall childbirth experience (*n* = 898). Five out of seven statements about perceptions of care during childbirth remained independently significant in the multivariable model: confidence in staff (adjusted OR 1.73, 95% CI 1.20–2.49), receiving enough information (adjusted OR 1.44, 95% CI 1.03–2.00), being treated with respect (adjusted OR 1.69, 95% CI 1.18–2.43), getting support from staff (adjusted OR 1.75, 95% CI 1.20–2.56), and having the baby skin-to-skin after birth (adjusted OR 2.21, 95% CI 1.52–3.19) (see Fig. 1). Area under the ROC curve for the final model was 0.79 (95% CI 0.75–0.82).

## Discussion

The majority of women who had given birth the last 1 to 13 months (77.5%) reported a good overall childbirth experience defined as a rating of 8–10 on a numeric rating scale from 0 (Very bad) to 10 (Very good) in response to the question: What was your overall experience of childbirth? Seven statements of perceptions of care were significantly related to a good overall experience in univariate analyses and five of them remained significant as independent predictors in a multivariable logistic regression model: having confidence in staff, receiving enough information during childbirth, being treated with respect, getting support from staff, and having the baby skin-to-skin after birth. The current results corroborate earlier findings [9, 11, 17–22] and also contribute to new knowledge that all these factors independently contribute to an overall good childbirth experience.

Having confidence in the skills of the staff was contributing to a good experience. This fact corroborates earlier findings that confidence in staff is central for the experience [9, 17, 18]. Furthermore, a lack of trust in the childbirth staff may lead to fear to give birth [23] and associated negative consequences [12]. Confidence and trust in staff is facilitated with continuity of care [24]. Continuity is defined as an indicator on good quality of care [25] and therefore can confidence in staff be seen as an indirect indicator on quality of care.

Having received enough information during childbirth was also an independent predictor for a good experience. This finding corresponds to earlier research where information has been identified as an important factor during labour and birth, where lack of communication and information has been shown as a reason for dissatisfaction both in Sweden [19] and in Tanzania [20]. A large observational study in five countries in south and east Africa showed insufficient communication and information to be part of disrespectful care and the authors draw the conclusion that quality of care in maternity care in low-income countries needs to be improved [7].

The next independent predictor, being treated with respect, is interlinked with trust and confidence in staff [9]. Insufficient communication and information is also previously shown as deficiencies in respectful care [7]. Being treated with respect is needed in building a trusting relationship and fundamental in high-quality care [7], where the relationship between the woman and the midwife is central in a theoretical model of women-centred care [26]. Interviews with women with secondary fear of childbirth showed that being treated with dignity was a main theme for a positive experience [27].

Getting support from the staff during childbirth was also shown to be an independent predictor of a good experience. This current piece of evidence adds to very thorough and convincing research evidence that continuous support is associated with maternal satisfaction and furthermore with increased chance of an uncomplicated vaginal birth, less use of medical pain relief methods during labour, shorter labour and a reduced risk for the baby to be born with a low Apgar score [21].

The fifth independent predictor of a good childbirth experience in this study was for the mother to get her newborn baby skin-to-skin after birth. This is shown in a Cochrane review to be a very important caring intervention to promote successful breastfeeding with no adverse effects for the mother or baby [28]. To have the baby skin-to-skin has also shown to increase maternal satisfaction [22].

### Strengths and limitations to the study

There are some limitations to this study. As a cross-sectional study, it is unable to draw causal inference. The women’s childbirth experience and their perceptions of care during childbirth were assessed 1 to 13 months postpartum but time was not considered in our analysis. Earlier studies have shown that women’s childbirth experience may change two to 5 years postpartum [15, 29, 30]. However, a longitudinal cohort study of more than 1000 women showed that the women recollected their birth memories clearly 5 years postpartum [31]. Another limitation is that, because data were collected in face-to-face interviews, the women may have been reluctant to express negative views about their experiences and their perceptions of the care they received. The interviewers had not been involved in the care of these women, thus reducing this risk. Strengths include that the large sample and the random selection of the study population makes it possible to generalise the study results to the whole population in Rwanda. Also, to our knowledge, this is the first study to investigate associations between perceived care and childbirth experience in Rwanda.

## Conclusions

In summary, the current results add to the evidence of women’s perceptions of childbirth care and how they relate to the overall childbirth experience. The independent predictors of a good experience were interlinked and are also useful indicators for care with good quality. To further improve childbirth care in Rwanda and care for women according to their preferences, it is important to make sure that childbirth care includes the following quality aspects in national and clinical guidelines: build confidence, provide good information, treat women and families with respect, provide good professional support during childbirth and put the newborn baby skin-to-skin with its mother early after birth.

## Abbreviations

CHW: Community Health Worker
MatHeR: Maternal Health Research Programme
